# Optimization and characterization of a lactate-oxidase electrode

**DOI:** 10.1039/d5ra07173a

**Published:** 2025-11-04

**Authors:** Ke Shi, Selvarajan Varshini, Keerthi Booshan Manikandan, Gyemin Lee, Chang-Joon Kim

**Affiliations:** a Department of Chemical Engineering and RIGET, Gyeongsang National University 501 Jinju-daero Jinju Gyeongnam 660-701 Republic of Korea cj_kim@gnu.ac.kr; b Department of Information and Statistics, Gyeongsang National University Jinju Republic of Korea

## Abstract

Electrochemical lactate sensors, used for analyzing biological fluids such as blood, sweat, and saliva, are gaining significant interest in healthcare and sports fields. A pivotal element that affects these sensors' performance is the lactate oxidase-based electrode. This study focused on enhancing the performance of the lactate oxidase electrode by optimizing the loading and layering of lactate oxidase (LOx) and poly(ethylene glycol) diglycidyl ether (PEGDGE) on carbon paper *via* the Box–Behnken design. The electrode surface was characterized utilizing FE-SEM, FTIR, and impedance analyses, with performance evaluation conducted *via* electrochemical and biochemical analyses. The optimized electrode, incorporating four layers of LOx (1.9 U) and PEGDGE (184 μg), generated an oxidation current of 1840 ± 60 μA, displaying high enzyme activity. It exhibited a maximum current at a lactate concentration of 50 mM, an apparent *K*^app^_m_ of 11.4 mM, and high stability with robust enzyme binding, thus supporting repeated use across numerous cycles. These results are instrumental in advancing the development of more effective and dependable lactate biosensors.

## Abstract 2

The performance of the lactate oxidase electrode was enhanced by optimizing the loading and layering of lactate oxidase and poly(ethylene glycol) diglycidyl ether on carbon paper.

## Graphical abstract

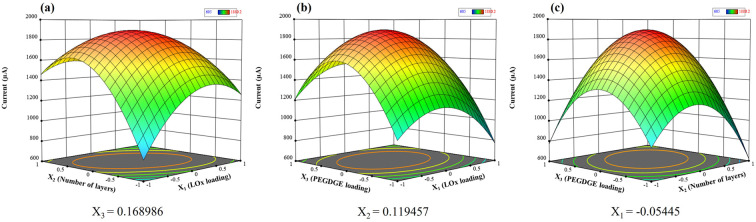

## 1. Introduction

Lactate levels in biological fluids such as blood, sweat, and saliva offer critical insights into metabolic states, exercise intensity, and conditions like sepsis, ischemia, and lactic acidosis.^1,2^ Electrochemical sensors based on lactate oxidase (LOx) have proven to be effective for real-time monitoring of lactate concentrations in both clinical and non-clinical settings.^1,2^ These sensors operate by catalyzing the oxidation of lactate to pyruvate through the LOx electrode, which results in the generation of a current that is proportional to the lactate concentration.^3^ The performance of lactate biosensors relies heavily on the proficient immobilization of lactate oxidase on the electrode surface.^4^ It is essential for the enzyme to remain securely attached to its carrier for prolonged periods while maintaining its activity.^5,6^

Polyethylene glycol diglycidyl ether (PEGDGE) is extensively utilized as a cross-linking agent for creating stable and biocompatible matrices for enzyme immobilization. The incorporation of PEGDGE ensures robust enzyme binding while preserving the enzyme's activity and stability over extended periods.^7^ Nonetheless, determining the optimal PEGDGE loading is essential, since inadequate loading leads to low biosensor sensitivity, whereas excessive loading diminishes enzyme activity.^7^ Furthermore, an increase in enzyme loading accelerates reaction rates until a critical threshold,^8,9^ after which, mass transfer limitations and augmented electron transfer resistance diminish performance.^10–14^ Consequently, meticulous optimization of PEGDGE and LOx loading and layering on the electrode surface is vital to maximize enzyme activity and stability, thereby directly enhancing the sensor's performance. To achieve optimum electrode performance, we employed response surface methodology (RSM), encompassing a suite of mathematical and statistical techniques designed for process development, improvement, and optimization. Specifically, the Box–Behnken design (BBD), a RSM variant, was adopted for its efficiency and practicability in experimental configuration, enabling the evaluation of the interactive effects of multiple variables with fewer experiments.^15^

This study aims to improve electrode performance by fine-tuning the loading and arrangement of LOx and PEGDGE on the electrodes. Employing the BBD, we analyzed the interactive effects of various factors on electrode response to establish optimal conditions for enhanced lactate detection. The optimized electrode was characterized using field-emission scanning electron microscopy (FE-SEM) and Fourier transform infrared (FTIR) analysis, and its performance was assessed through electrochemical and biochemical methods. The insights from this research will aid in developing more effective and reliable lactate biosensors.

## 2. Experimental methods

### 2.1 Chemicals

A LOx from *Aerococcus viridans* (50 U, 45 units per mg solid, product no. L9795), peroxidase from horseradish (5 KU, product no. P8250), PEGDGE (average molecular weight ≅500), sodium chloride (NaCl), disodium hydrogen phosphate (Na_2_HPO_4_), potassium chloride (KCl), potassium dihydrogen phosphate (K_2_HPO_4_), sodium l-lactate (98%), phenazine methosulfate (PMS), 4-aminoantipyrine (4-AAP), *N*,*N*′-dimethylaniline (DMA), dodecylbenzenesulfonic acid (DBS), flavin adenine dinucleotide (FAD), and other chemicals were procured from Sigma-Aldrich (MO, USA). Polyvinyl chloride (PVC) film with a thickness of 200 μm was obtained from Office Depot (Seoul, Korea). Hydrophilic-type carbon paper (CP, product no. HCP-020N) was sourced from WizMAC (Seoul, Korea) with the following specifications provided by the supplier: thickness, 0.2 ± 0.01 mm; density, 0.78 g cm^−3^; resistivity, 2.5 mΩ cm^2^; and porosity, 75%.

### 2.2 Fabrication of enzyme electrode

Due to the weak mechanical properties of carbon paper, a PVC film was attached to one side of a 3M double-sided adhesive tape, utilizing a transparent film as the support matrix. On the opposite side of the tape, a CP (3 cm × 0.3 cm), precisely cut to the required dimensions, was affixed. The lower (0.5 cm × 0.3 cm) and upper parts of the CP were exposed for enzyme immobilization and connection to a potentiostat, respectively.

Lyophilized LOx was dissolved in 10 mM Phosphate-Buffered Saline (PBS) solution (pH 7.0) containing 10% glycerol to prepare a highly concentrated solution, which was then stored in a freezer at −20 °C. The 10 mM PBS buffer is composed of 137 mM NaCl, 10 mM Na_2_HPO_4_, 2.7 mM KCl, and 2 mM K_2_HPO_4_. The stock solution was diluted with distilled water to achieve the desired LOx concentration. This solution was mixed with PEGDGE in a volume ratio of 4 : 1 to form a LOx–PEGDGE mixture. A 20 μL aliquot of this mixture was applied to the surface of the CP and allowed to dry at room temperature for 2 hours. To fabricate electrodes with multiple layers of LOx–PEGDGE, a layer-by-layer adsorption technique was employed.

### 2.3 Experimental design for the optimal fabrication of enzyme electrodes

The BBD was used to optimize the loadings of LOx and PEGDGE, as well as to determine the optimal number of LOx–PEGDGE layers on the surface of CP. Based on preliminary studies, the experimental ranges for these three key factors were established, leading to a total of seventeen experiments, inclusive of five center-point runs, to develop response surface models (Table 1).

**Table 1:** Box–Behnken design for the optimization of three independent variables^a^

Run	*X*_1_		*X*_2_		*X*_3_		*Y*	
	Coded	Uncoded (U)	Coded	Uncoded (no)	Coded	Uncoded (μg)	Experiment (μA)	Calculation (μA)
1	+1	4	+1	7	0	160	893	911
2	+1	4	−1	1	0	160	1265	1278
3	−1	0.08	+1	7	0	160	1437	1424
4	−1	0.08	−1	1	0	160	838	820
5	+1	4	0	4	+1	300	1164	1228
6	+1	4	0	4	−1	20	923	828
7	−1	0.08	0	4	+1	300	1066	1161
8	−1	0.08	0	4	−1	20	1014	950
9	0	2.04	+1	7	+1	300	1413	1331
10	0	2.04	+1	7	−1	20	528	605
11	0	2.04	−1	1	+1	300	869	792
12	0	2.04	−1	1	−1	20	823	906
13	0	2.04	0	4	0	160	1957	1880
14	0	2.04	0	4	0	160	1821	1880
15	0	2.04	0	4	0	160	1866	1880
16	0	2.04	0	4	0	160	1830	1880
17	0	2.04	0	4	0	160	1927	1880

a *X*_1_, the LOx loading (U); *X*_2_, the number of LOx–PEGDGE layers; *X*_3_, the PEGDGE loading (μg).

Regression analysis of the experimental data was performed using SAS software for Windows (version 9.4, SAS Institute Inc., Cary, NC, USA), and the optimal levels of the three factors were identified by solving a second-order polynomial equation and reviewing the response surface plots:*Y* = *β*_0_ + ∑*β*_*i*_*X*_*i*_ + ∑*β*_*ii*_*X*_*i*_^2^ + ∑*β*_*ij*_*X*_*i*_*X*_*j*_,where *Y* is the predicted response, *β*_0_ is the offset term, *β*_*i*_ is the linear effect, *β*_*ii*_ is the squared effect, *β*_*ij*_ is the interaction effect, and *X*_*i*_ and *X*_*j*_ are the dimensionless coded values of the variable *x*_*i*_ and *x*_*j*_ respectively. The correlation between the coded values and their actual values is expressed by the equation:
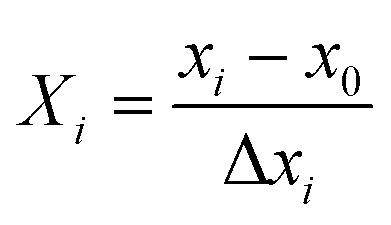
where *X*_*i*_ is the dimensionless (coded) value of the independent variable *x*_*i*_; *x*_0_ represents the actual value of *x*_*i*_ at the center point, and Δ*x*_*i*_ denotes the step change of *x*_*i*_.

The response surface curve was generated using Design Expert (version 23.1, Stat-Ease 360 Inc., Minneapolis, USA), based on the polynomial equation derived from the regression analysis performed in SAS. While analyzing the effect of two factors, the third factor was kept constant.

### 2.4 Surface characterizations and biochemical analyses of the electrodes

The surface morphology of the modified CP was examined using FE-SEM (Apero 2S, Thermo Fisher Scientific) coupled with energy-dispersive X-ray spectroscopy (EDS, Oxford Instrument, Aztec Software) at an accelerating voltage of 20 kV. Before analysis, all samples were mounted on carbon tape and coated with Pt using a sputter coater (208HR, Cressington Scientific Instruments, UK), achieving a coating thickness of approximately 0.1–10 nm under a vacuum pressure of 0.1 mbar.

The FTIR spectra of both the bare and enzyme-modified electrodes were recorded using a Vertex 80 V FTIR spectrometer (Bruker, USA) equipped with a single-reflection diamond attenuated total reflectance (ATR) accessory, ATR Special Quest. The samples were placed directly into the sample cell to ensure effective contact, enabling the acquisition of high-quality infrared spectra non-destructively. The spectra were recorded over a wavenumber range of 4000 to 400 cm^−1^. The derived spectra offered valuable insights into the subtle structural characteristics of the materials under investigation. The spectral data analysis was conducted using OMNIC™ spectra software.

The activity of LOx was assessed using a coupled enzyme assay, adapted from a protocol provided by Asahi Kasei Corporation (Tokyo, Japan). In this assay, LOx catalyzes the oxidation of lactate, leading to the production of hydrogen peroxide (H_2_O_2_). The resulting H_2_O_2_ interacts with 4-AAP and DMA in a reaction catalyzed by peroxidase, forming a red-violet quinoneimine.^16^ The rate of quinoneimine formation, which correlates directly with the lactate concentration, was measured colorimetrically at 565 nm using a UV/Vis spectrophotometer (Shimadzu, Japan).

One milliliter of the reaction mixture, comprising 0.2 mL of potassium phosphate buffer (0.2 M, pH 6.5), 0.1 mL of peroxidase solution (50 U mL^−1^), 0.1 mL of 4-AAP solution (15 mM), 0.1 mL of sodium lactate solution (0.5 M, pH 6.5), 0.2 mL of DMA solution (0.2%, w/v), and 0.3 mL of distilled water, was transferred into a 15 mL Falcon tube and preincubated at 37 °C. After 5 minutes, the reaction was initiated by adding 20 μL of LOx solution (1 mg mL^−1^) for the free enzyme system, or by inserting carbon paper immobilized with LOx and PEGDGE, along with 20 μL of buffer solution to maintain the volume. For the test blank, 20 μL of potassium phosphate buffer replaced the LOx solution. The mixture was then incubated in a shaking incubator (Jeio Tech, Seoul, Korea) at 37 °C and 180 rpm. After ten minutes, the reaction was halted by adding 2 mL of DBS solution (0.2% v/v), and the absorbance at 565 nm (*A*_565_) was measured. The reaction mixtures were diluted if the change in absorbance (Δ*A*_565_) exceeded 0.350. One enzyme unit is defined as the quantity of enzyme that produces 1 μmole of H_2_O_2_ per minute at 37 °C under the specified assay conditions. Enzyme activity was calculated using the following equation:
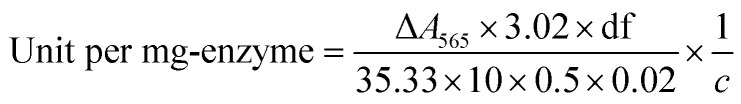
where Δ*A*_565_ represents the difference between *A*_sample_ and *A*_blank_ at 565 nm. 3.02: total volume of assay (mL). df: dilution factor. 35.33: millimolar extinction coefficient of quinoneimine dye (L mmol^−1^ cm^−1^). 0.5: conversion factor based on one mole of H_2_O_2_ producing half a mole of quinoneimine dye. 10: reaction time. 0.02 mL = volume of enzyme solution. *c* represents the concentration of enzyme in the solution (mg mL^−1^).

### 2.5 Electrochemical analysis of enzyme electrodes

The electrochemical cell was a three-electrode system comprising a working electrode (CP–LOx–PEGDGE), a counter electrode (Pt), and an Ag/AgCl reference electrode (LF-2, Innovative Instruments Inc., Tampa, FL, USA). The electrolyte consisted of 10 mL of 10 mM PBS (pH 7.0) with 1 mM PMS and 50 mM sodium l-lactate, unless otherwise specified. To deoxygenate the electrolyte, nitrogen gas was bubbled through the solution for 10 minutes prior to use. Cyclic voltammetry (CV) was performed at a scan rate of 25 mV s^−1^ over a potential range of −0.6–0.6 V (*vs.* Ag/AgCl). In the chronoamperometry (CA) tests, a steady oxidation potential of −0.05 V (*vs.* Ag/AgCl) was applied. The working electrode was equilibrated in an electrolyte devoid of lactate for 15 minutes before the electrochemical test commenced with the addition of lactate. Electrochemical impedance spectroscopy (EIS) measurements were performed using 10 mM PBS (pH 7.0) containing 5 mM K_3_ [Fe(CN)_6_]/K_4_ [Fe(CN)_6_] (1 : 1) mixture as a redox probe at the charged state of 0.2 V in the frequency range of 0.1 to 100 000 Hz with a potential perturbation of 5 mV. The reusability of the optimized electrode was evaluated by replacing the lactate-containing electrolyte solution (50 mM) with a fresh one after each cycle during a CA experiment. All electrochemical measurements were carried out at room temperature using an electrochemical workstation (CH Instruments, model 660D, Houston, TX, USA).

## 3. Results and discussion

### 3.1 Selection of key factors and determination of their testing ranges

**Fig. 1:**
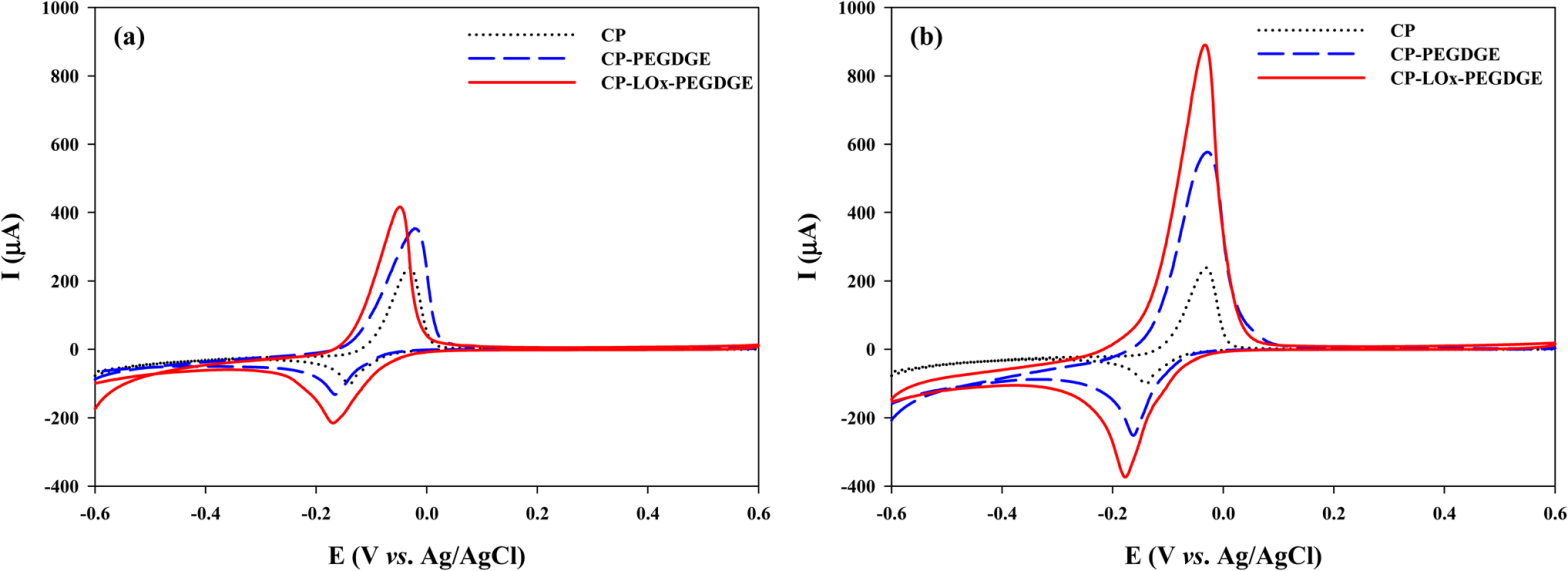
Cyclic voltammograms of CP, CP–PEGDGE, and CP–LOx–PEGDGE in an electrolyte (a) without and (b) with 50 mM lactate. The buffer was 10 mM PBS (pH 7.0) supplemented with 1 mM PMS. LOx and PEGDGE loadings were 0.3 U and 40 μg, respectively.

Cyclic voltammograms of CP, CP–PEGDGE (40 μg), and CP–PEGDGE (40 μg)–LOx (0.3 U) electrodes were recorded in electrolyte containing 1 mM PMS, both in the absence (Fig. 1(a)) and presence of 50 mM lactate (Fig. 1(b)). Oxidation and reduction peaks were observed at −0.05 and −0.17 V (*vs.* Ag/AgCl), respectively, corresponding to the oxidation–reduction characteristics of PMS.^17^ In the absence of lactate, all electrodes exhibited nearly identical peak positions, indicating that PEGDGE or LOx attachment did not significantly affect the redox peak potential. In the presence of lactate, the CP–PEGDGE–LOx electrode displayed a pronounced increase in peak current relative to bare CP, consistent with LOx-catalyzed oxidation of lactate to pyruvate with concomitant electron generation, while the peak potential remained unchanged. These results suggest that PEGDGE primarily serves as an enzyme immobilization matrix, while PMS efficiently mediates electron transfer regardless of surface modification.^18,19^

Generally, the amount of enzyme loaded on the electrode profoundly influences its performance, where increased enzyme concentration boosts conversion rates as previously discussed. However, excessive enzyme loading can lead to mass transfer limitations and hamper electron transfer.^20^ To achieve optimal physical stability of the enzyme electrode, strong adhesion of the enzyme to the electrode surface is crucial since weakly bound enzymes are prone to desorption.^21^ PEGDGE, with its two reactive epoxy groups, can interact with amino, hydrogen, and carboxylic groups. Its reaction with amine groups in proteins creates a matrix that facilitates the immobilization of the enzyme on the electrode surface, thereby enhancing attachment strength through the interconnected enzyme network formed by PEGDGE.^7^ Nevertheless, the activity and stability of the immobilized enzyme are influenced by the amount of PEGDGE used. Therefore, investigations were conducted on the effects of varying LOx and PEGDGE loadings.

**Fig. 2:**
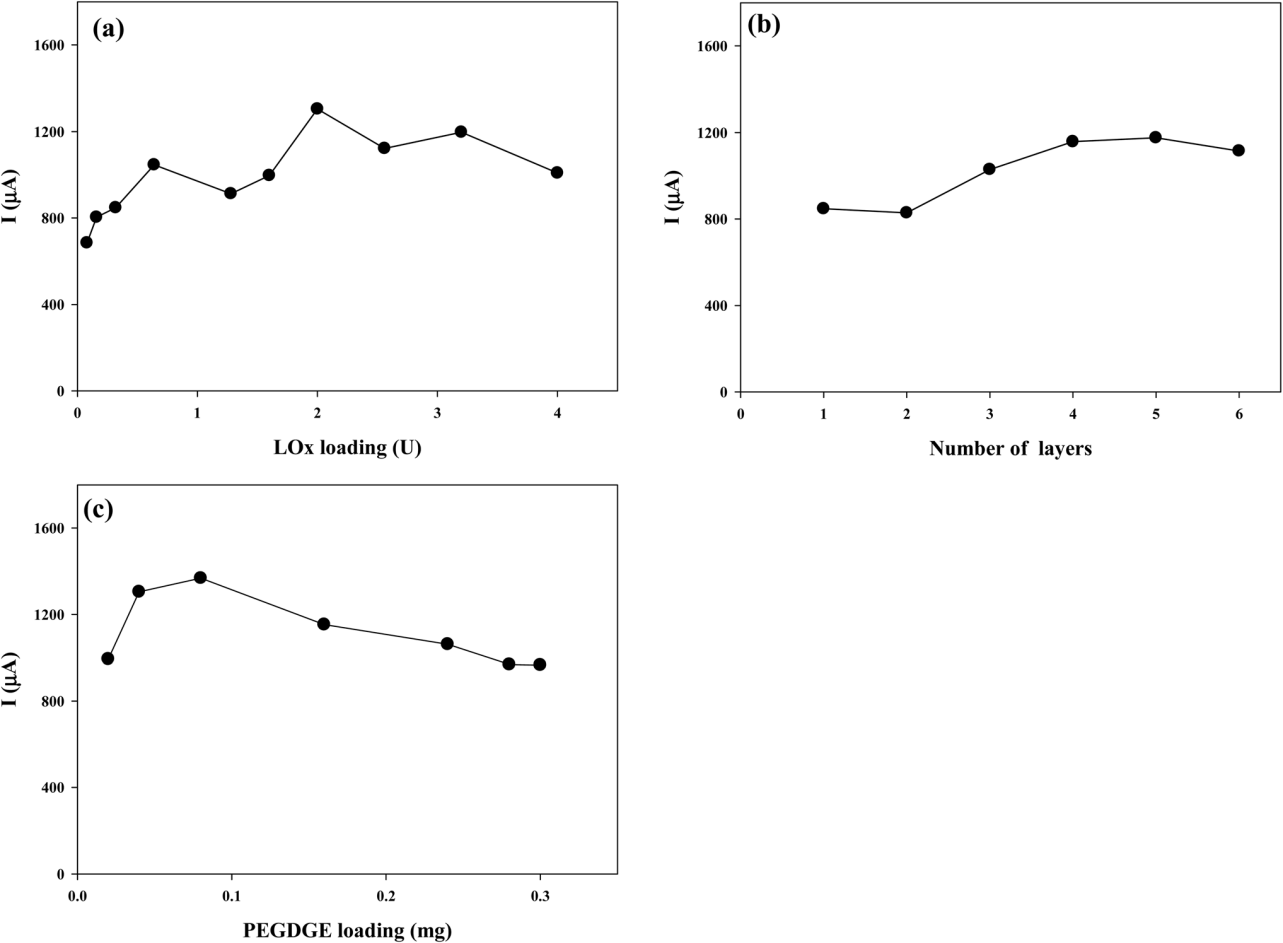
The effect of (a) LOx loading, (b) number of layers, and (c) PEGDGE loading on the peak oxidation current generation of CP–LOx–PEGDGE in 10 mM PBS buffer (pH 7.0) containing 1 mM PMS and 50 mM lactate.

As a preliminary experiments, the individual effect of each variable (LOx loading, PEGDGE loading, and LOx–PEGDGE layer) was investigated using a one-factor-at-a-time approach. The electrodes were fabricated by varying LOx loading while maintaining PEGDGE loading at 40 μg. The electrode performances were evaluated by comparing the intensity of peak oxidation currents around −0.05 V (*vs.* Ag/AgCl). Fig. 2(a) illustrates the peak currents generated by the CP–LOx–PEGDGE electrode with different LOx loadings. As the LOx loading increased, the current generation also increased, reaching a maximum of 1300 μA at 2 U of LOx on CP. The peak current also varied with different numbers of LOx–PEGDGE layers. As the number of immobilized layers of LOx (0.32 U)–PEGDGE (40 μg) increased, there was an increase in current generation. The highest current (1200 μA) was observed at four layers of LOx–PEGDGE (Fig. 2(b)). We further analyzed the impact of PEGDGE loading on the CP–LOx–PEGDGE electrode performance, where LOx loading was fixed at 2 U, and PEGDGE loading was adjusted. Experimental results revealed that the maximum current of 1400 μA was generated with a PEGDGE loading of 80 μg, as demonstrated in Fig. 2(c). Beyond this loading, the current generation declined. Excessive PEGDGE loading results in increased gel thickness, which impedes substrate transfer from the solution, leading to reduced enzyme activity; however, the enzyme may be more stably immobilized on the electrode surface.^7^

The results indicate that the highest current is achievable only when the LOx loading, PEGDGE loading, and number of LOx–PEGDGE layers are optimized. In this experiment, however, other factors remained constant, and only one factor was manipulated at a time. This approach does not consider the interactive effects among the variables studied, thus not fully representing the overall impact of the factor on the response throughout the entire factor space.^22^ Nevertheless, the results provide a valuable basis for setting the experimental range using the BBD.

### 3.2 Determining the optimal condition of LOx, PEGDGE, and LOx–PEGDGE layers

Since the LOx loading, PEGDGE loading, and the number of LOx–PEGDGE layers significantly influence the performance of the electrode, these three factors were optimized using BBD. This strategy was employed not only to explore the response across the entire factor space but also to pinpoint the region where the response achieves its optimal or near-optimal value.^15^

Table 2 presents the calculated coefficients and their respective significance levels. *P* values are utilized to determine the statistical significance of each coefficient, offering insights into the interplay among variables. A smaller *P* value suggests a stronger significance of the corresponding coefficients.^23,24^ The quadratic coefficients for *x*_1_^2^, *x*_2_^2^, and *x*_3_^2^ showed high significance (*P* = 0.0001), while the linear coefficient for *x*_3_ and the interaction coefficients for *x*_1_*x*_2_ and *x*_2_*x*_3_ were significant (*P* = 0.004). In contrast, the coefficients for other terms (*x*_1_, *x*_2_, *x*_1_*x*_3_) were not significant. Through multiple regression analysis of the experimental data, the relationship between the response variable and the independent variables was expressed by the following second-order polynomial equation:*Y* = 1.8802 − 0.0137*X*_1_ + 0.0595*X*_2_ + 0.1530*X*_3_ − 0.3192*X*_1_^2^ − 0.4527*X*_2_^2^ − 0.5192*X*_3_^2^ − 0.2428*X*_1_*X*_2_ + 0.0473*X*_1_*X*_3_ + 0.2098*X*_2_*X*_3_,where *Y* represents the amount of current generated by the CP–LOx–PEGDGE electrode, and *X*_1_, *X*_2_, and *X*_3_ indicate the LOx loading, number of LOx–PEGDGE layers, and PEGDGE loading, respectively.

**Table 2:** Variance analysis of the regression model

Source	Sum of squares	Df	Mean squares	*F*-Value	*p*-Value
Model	3.3358	9	0.3706	38.62	0.0000
*X*_1_	0.0015	1	0.0015	0.16	0.7032
*X*_2_	0.0283	1	0.0283	2.95	0.1295
*X*_3_	0.1873	1	0.1873	19.51	0.0031^a^
*X*_1_ *X*_2_	0.2357	1	0.2357	24.56	0.0016^a^
*X*_1_ *X*_3_	0.0089	1	0.0089	0.93	0.3669
*X*_2_ *X*_3_	0.1760	1	0.1760	18.34	0.0036^a^
*X*_1_^2^	0.5900	1	0.5900	61.47	0.0001^a^
*X*_2_^2^	0.9730	1	0.9730	101.39	0.0000^a^
*X*_3_^2^	1.1351	1	1.1351	118.28	0.0000^a^
Residual error	0.0672	7	0.0096		
Lack of fit	0.0528	3	0.0176	4.92	0.0788
Pure error	0.0143	4	0.004		
Cor. total	3.403	16			
Significance level	0.05				
*R* ^2^ = 0.9797, Adj. *R*^2^ = 0.9537, coefficient of variation = 7.668%					

a Significantly valued.

An analysis of variance (ANOVA) was conducted to determine the significance of the model. The technique was used to evaluate the statistical significance of the ratio of the mean square regression to the mean square residual error. The coefficient of determination (*R*^2^) for the quadratic regression model was 0.9797, indicating that the model accounts for approximately 98% of the variance in the response variable, suggesting a strong fit. The robustness of the model was further validated by the adjusted coefficient of determination (Adj. *R*^2^ = 0.9537), which compensates for the number of predictors in the model. Moreover, the *F*-statistic was 38.62, with a probability value of *P* < 0.0001, highlighting that the regression model significantly explains the variation in the response variable and supports the rejection of the null hypothesis that all regression coefficients are equal to zero.^25^ Additionally, the lack-of-fit *F*-value stood at 4.45, with an associated *P* value of 0.079. Since this *P*-value is above the standard significance threshold of 0.05, the lack-of-fit is considered statistically insignificant, further affirming that the model satisfactorily fits the data.^26^ Therefore, the regression model is considered reliable for predicting the response variable. The predicted results obtained from the empirical model are detailed in Table 1.

**Fig. 3:**
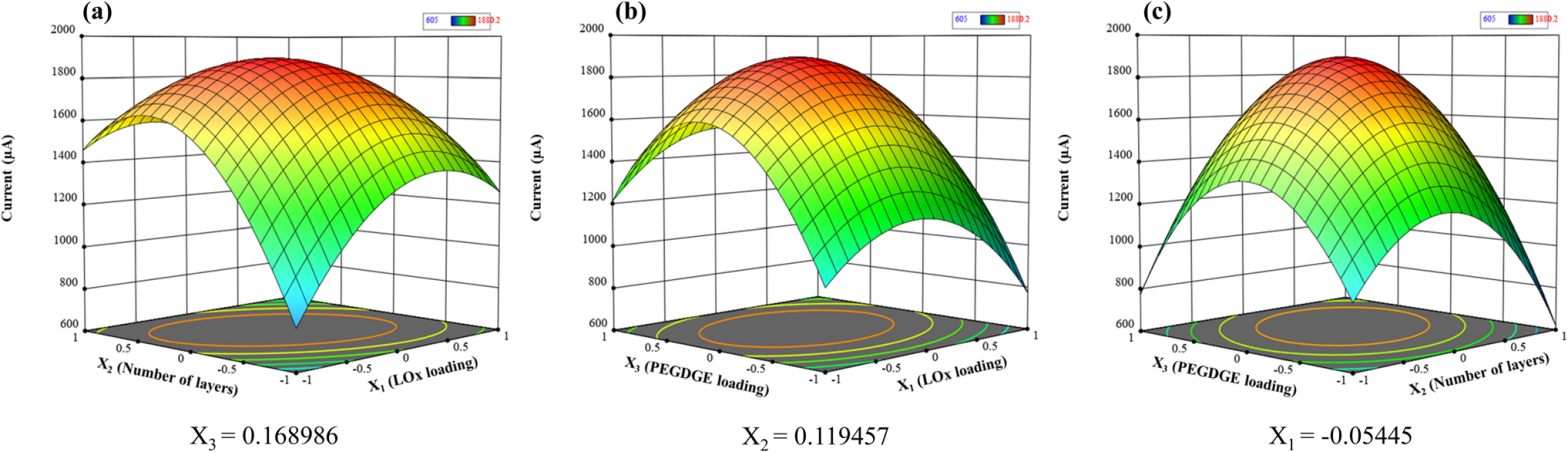
Response surface plots showing the combined effects of (a) LOx loading and the number of LOx–PEGDGE layers, (b) LOx and PEGDGE loadings, and (c) PEGDGE loading and the number of LOx–PEGDGE layers on the oxidation current, obtained under 10 mM PBS buffer (pH 7.0) supplemented with 10 mM PMS and 50 mM lactate.

Based on the predicted model, 3D response surface plots were generated to visualize the effects of various factors. Fig. 3(a) illustrates the impact of LOx loading and the number of LOx–PEGDGE layers on electrode performance. An increase in LOx–PEGDGE layers results in higher current generation by the CP–LOx–PEGDGE electrode. As the number of layers approaches the center point, the current generated by the electrode reaches a maximum of 1897 μA, which is 2.2 times greater than that produced by a single LOx–PEGDGE layer. However, if the LOx–PEGDGE layers continue to increase beyond the center point, the current does not increase proportionally. Fig. 3(b) and (c) show that both LOx loading and PEGDGE loading simultaneously impact electrode performance, with concurrent influences from LOx–PEGDGE layers and PEGDGE loading.

The model equation demonstrates interactions among the three factors, illustrating that maximum peak current is achievable near the center point. Accordingly, the optimal conditions for attaining peak electrode performance (1897 μA) were identified near this center point. The equation derived from the SAS software provides the coded optimal values for the three factors (*X*_1_, *X*_2_, and *X*_3_) as follows: *X*_1_ = −0.05445, *X*_2_ = 0.119457, and *X*_3_ = 0.168986. These values correspond to *x*_1_ = 1.9 U of LOx, *x*_2_ = four layers of LOx–PEGDGE, and *x*_3_ = 184 μg of PEGDGE. Thus, the optimized electrode surface is covered with four layers of LOx (1.9 U)–PEGDGE (184 μg), denoted as CP–(LOx–PEGDGE)_4_. To validate the simulation result, five CP–(LOx–PEGDGE)_4_ electrodes were fabricated under these optimal conditions, and their performance was assessed using CV across five repeated experiments. The peak current generated by the electrodes was 1840 ± 60 μA, closely matching the simulation results. This corresponds to the calculated current density of 12.3 ± 0.4 mM cm^−2^ based on the geometric area of bare electrode (0.15 cm^2^).

### 3.3 Surface morphologies of optimized electrode

**Fig. 4:**
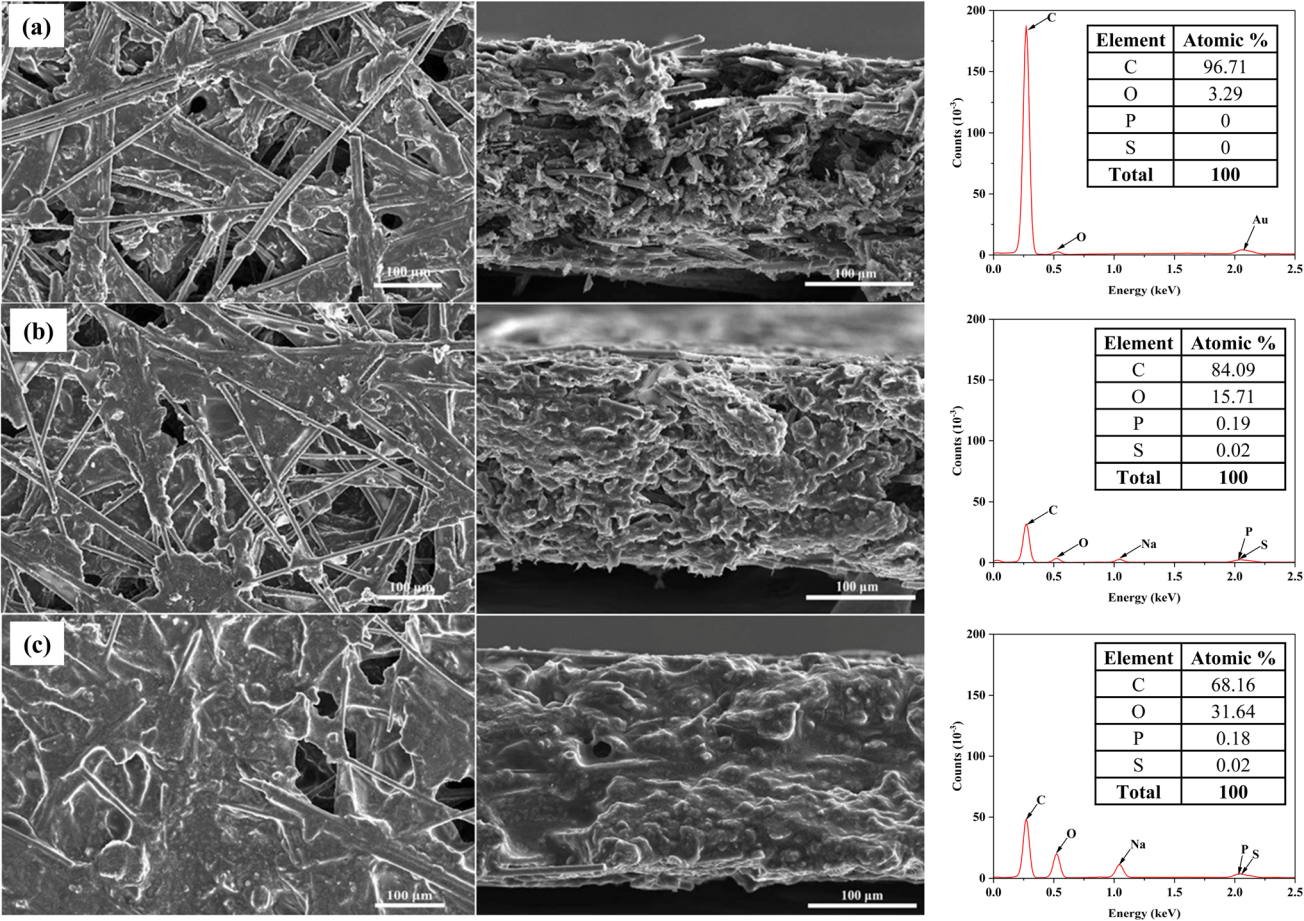
FE-SEM images and EDS mappings of the tops and cross sections of (a) CP, (b) CP with one layer of LOx–PEGDGE, and (c) CP with four layers of LOx–PEGDGE, representing the optimized electrode.

Fig. 4 presents SEM images and EDS spectra illustrating the surface morphology and compositional evolution of bare CP and CP covered with varying layers of LOx–PEGDGE. In the SEM images, bare CP displays interconnected carbon fibers and randomly distributed carbon flakes, evident both on its surface and within its interior, which contribute to its porous structure. After immobilization of LO–PEGDGE, the composite becomes entrapped within the pores of the CP. In the case of single LOx–PEGDGE layer, uncoated surface areas within the pores were observed, indicating incomplete substrate coverage. When CP is covered with four layers of LOx–PEGDGE, these pores are nearly filled, giving the surface a film-covered appearance.

The EDS spectrum of bare CP shows a dominant carbon (C) peak at ∼0.28 keV and a smaller oxygen (O) peak near 0.53 keV, with corresponding quantitative values of 96.70% C and 3.29% O (Fig. 4(a)). No phosphorus (P) or sulfur (S) peaks are detected; instead, distinct gold (Au) peaks ∼2.12 keV (Au Mα) arise from the thin gold coating applied to improve conductivity and prevent charging during SEM/EDS analysis.^27^ The morphology and composition of bare CP are consistent with previously reported findings.^28^ In CP immobilized with a single layer of LOx–PEGDGE, additional peaks corresponding to P (∼2.01 keV) and S (∼2.31 keV) emerge, accompanied by an increase in oxygen content to 15.71% (Fig. 4(b)). This rise in oxygen is attributed to oxygen-rich ether (C–O–C) and epoxy functionalities in PEGDGE, as well as oxygen-containing residues in LOx. Trace amounts of P (0.19%) and S (0.02%) likely originate from sulfur-containing amino acids in LOx and the phosphate-containing cofactor flavin mononucleotide (FMN). Notably, the Au peaks observed in (a) are absent in (b), which is likely due to the coverage of the gold-coated CP surface by the LOx–PEGDGE layer. This overlayer attenuates or completely blocks the Au X-ray emission from the underlying coating, making it undetectable by EDS. When four LOx–PEGDGE layers are applied, the intensities of the P and S peaks are further increased, while the carbon content decreases markedly to 68.16% and oxygen content increases to 31.64% (Fig. 4(c)). This compositional shift reflects the substantial accumulation of the oxygen-rich LOx–PEGDGE composite and near-complete coverage of the CP surface. The absence of Au peaks in (c) can be similarly explained by the even thicker LOx–PEGDGE attachment, which further suppresses X-ray signals from the gold layer beneath. The progressive enhancement of P and S peaks from (a) to (c) provides direct evidence for the stepwise and controllable immobilization process. A sodium peak was detected in CPs modified with LOx–PEGDGE, whereas no such peak appeared in bare CP. This signal is presumed to originate from the PBS used to dissolve LOx and was therefore excluded from EDS quantitative analysis. Nitrogen was not detected in any sample, most likely due to its low X-ray emission energy (∼0.392 keV), weak signal intensity, peak overlap with C and O, and the inherently low nitrogen content of the protein layers, making SEM/EDS detection particularly challenging.^29^

**Fig. 5:**
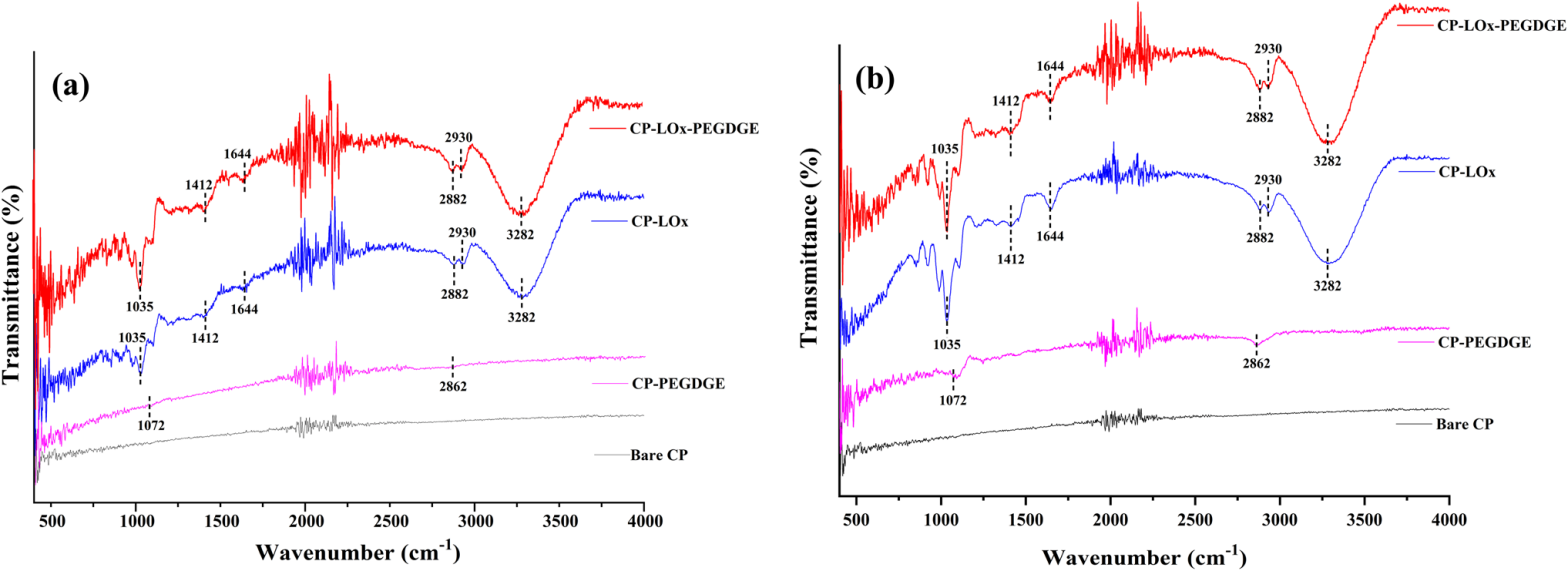
FTIR spectra of CP and CP coated with (a) one layer and (b) four layers of PEGDGE, LOx, and LOx–PEGDGE.

The FTIR analysis was conducted to investigate the presence of enzymes on the electrode surface. Fig. 5 presents the FTIR spectra of CP and CP covered with one or four layers of PEGDGE, LOx, or LOx–PEGDGE. Notable transmittance occurred within the functional group region of the spectrum (1000–4000 cm^−1^). Clear peak differences were visible in the spectra of CP and enzyme-immobilized CP, with the spectra containing four layers of enzyme showing more pronounced peaks than those with a single layer. Compared to the CP spectrum, the FTIR spectra of CP–PEGDGE reveal a new peak at 1072 cm^−1^, associated with the C–O and C–O–C stretching vibrations of PEGDGE.^30–32^ Additionally, a significant peak at 2862 cm^−1^ relates to the C–H stretching vibration of the methylene group in PEGDGE.^30,32^

In the CP–LOx spectrum, prominent peaks around 1035 cm^−1^ are attributed to C–O–C stretching vibrations in the carboxylic moieties of amino acids.^33^ Peaks at 1412 cm^−1^ and 1644 cm^−1^ are likely associated with the amide I band, which predominantly arises from C=O stretching vibrations in the peptide bonds of proteins.^33,34^ The strong peak at 3282 cm^−1^ is attributable to N–H stretching, indicative of the amide linkage formed by the enzyme.^35^ Meanwhile, the bimodal peak at 2882 and 2930 cm^−1^ originates from C–H stretching.^28^ These results conclusively demonstrate the successful immobilization of LOx and PEGDGE on the CP surface.

### 3.4 Biochemical and electrochemical impedance analysis of optimized electrode

**Fig. 6:**
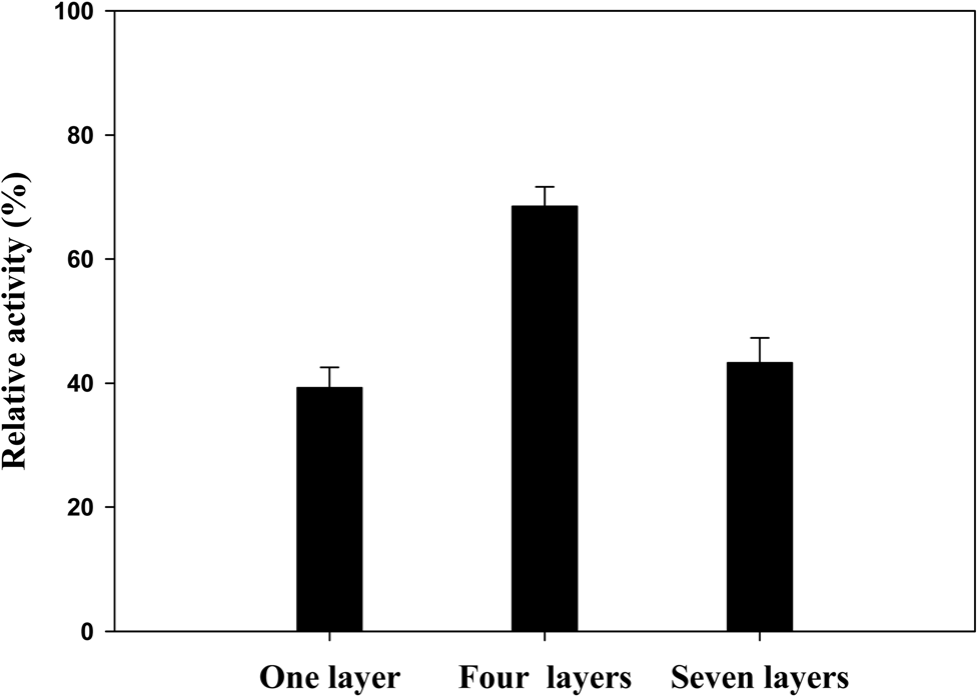
Comparison of the relative activities of CP coated with a single layer, four layers (representing the optimized electrode), and seven layers of LOx–PEGDGE.

To assess the impact of optimization on the activity of enzyme electrodes, the relative activity was measured by calculating the ratio of the specific activity of the immobilized enzyme to that of a control sample containing 20 μg of free enzyme, equivalent to 0.1 U. This investigation involved evaluating the relative activities of CP with varying layers of LOx–PEGDGE: a single layer, four layers (identified as the optimal configuration), and seven layers. Each layer consisted of 1.9 U of LOx and 184 μg of PEGDGE. It was observed that the immobilized enzyme exhibited lower relative activity compared to the free enzyme, likely due to mass transfer limitations within the immobilized enzyme systems.^5^ Notably, the electrode with four layers of LOx–PEGDGE demonstrated enhanced relative activity (68.5%) compared to the single-layer counterpart (39.3%), while the seven-layer electrode exhibited a slightly increased activity (43.8%) compared to the single-layer configuration (Fig. 6). This finding suggests that increasing the number of layers up to four enhances the lactate oxidation rate; however, adding more layers reduces the rate due to hindered diffusion of lactate through the thicker layers, which impedes its access to the immobilized LOx. This observation is consistent with the established principle that lactate-to-pyruvate conversion intensifies with higher LOx loading, but excessive layering restricts substrate diffusion and prevents the participation of all enzyme molecules in catalysis reaction.^11,13,20,36,37^

**Fig. 7:**
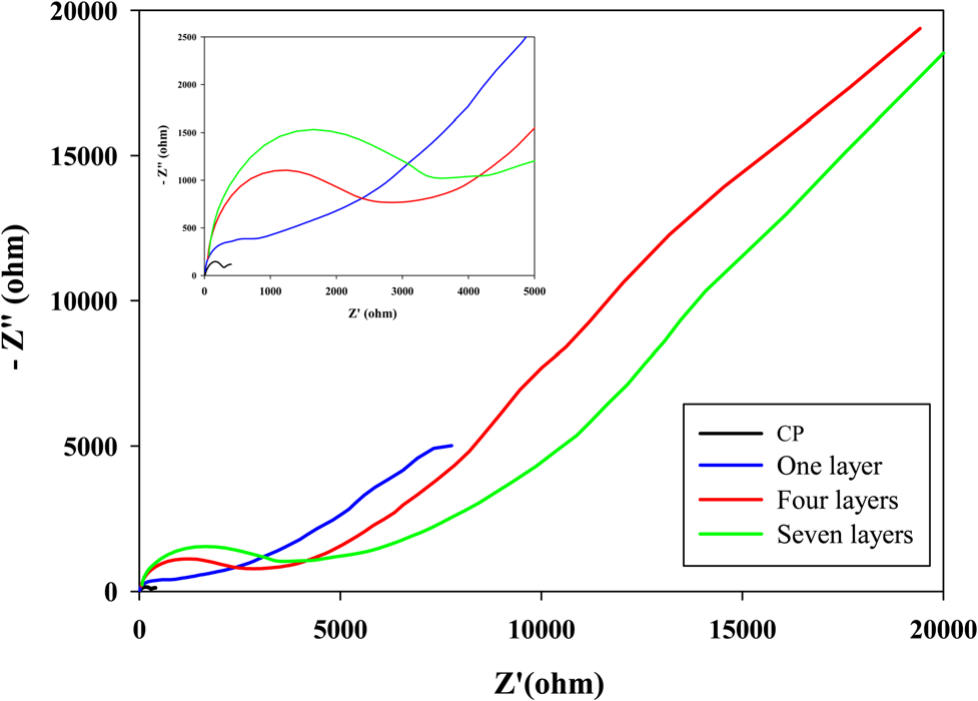
Nyquist plots of CP and CP coated with a single layer, four layers, and seven layers of LOx–PEGDGE. The inset shows an expanded view of the high-frequency region.

The EIS was subsequently employed to investigate the conductive properties of CP with different numbers of LOx–PEGDGE layers, using Fe(CN)_6_^3−/4−^ as a redox probe. Nyquist plots (Fig. 7) for bare CP and CP with one, four, and seven layers exhibited characteristic Randles-type behavior, consisting of a semicircle in the high-to-medium frequency region and a diffusion-related tail at low frequencies.^38^ This confirms that both charge-transfer and diffusion processes contribute to the overall electrochemical response. The high-frequency intercepts, corresponding to the solution resistance (*R*_s_), remained nearly identical across electrodes, reflecting consistent electrolyte and cell conditions.^38^ In contrast, the semicircle diameter, indicative of charge-transfer resistance (*R*_ct_), increased progressively with the number of layers, with the seven-layer electrode exhibiting an excessively large *R*_ct_. The one-layer electrode displayed a distorted, non-ideal semicircle, suggesting heterogeneous surface coverage and incomplete film formation, leaving portions of the CP substrate exposed.^39,40^ Such irregularity complicates charge-transfer pathways and raises concerns regarding reproducibility in electrode fabrication. At low frequencies, diffusion-related features also varied: bare CP showed a typical Warburg response, whereas the diffusion tail diminished with increasing layer numbers, evolving into a finite-length Warburg element in seven-layer electrode. This transition indicates that ion transport becomes progressively restricted within the multi-layered matrix, thereby reducing electrode responsiveness under operational conditions.

Taken together, these findings underscore the inherent trade-off between enzyme loading and diffusion/charge transport.^41–43^ Excessive enzyme loading not only hinders substrate access but also decrease electron conductance at the electrode surface.^44,45^ Therefore, it is essential to optimize enzyme loading carefully to strike a balance, maximizing the reaction rate while minimizing any adverse impact on electron transfer resistance. The four-layer electrode appears to represent a compromise, balancing catalytic activity with acceptable charge/electron-transfer resistance.

### 3.5 Lactate-sensing performance of the optimized electrode

**Fig. 8:**
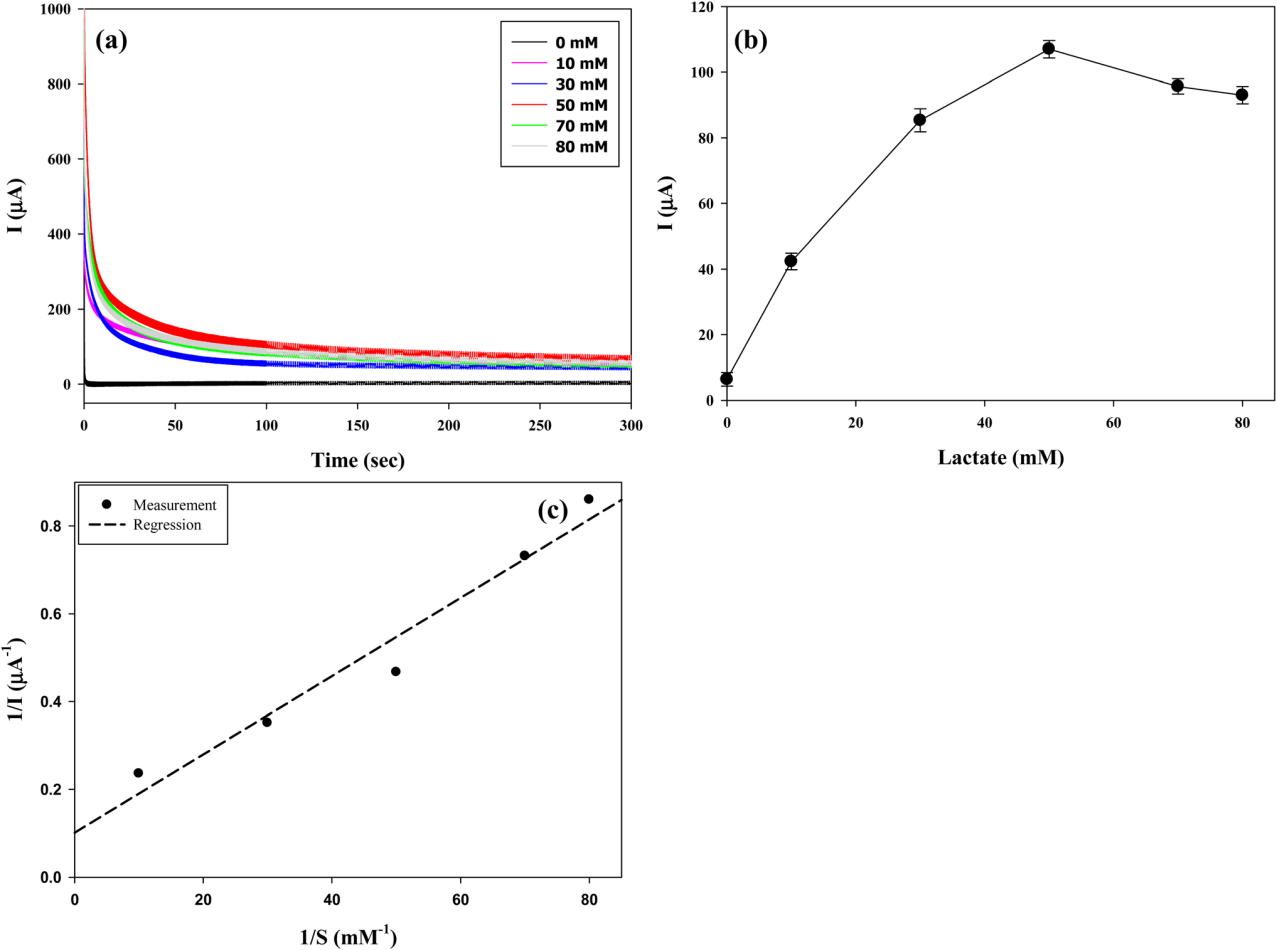
(a) Chronoamperometric current response over time at −0.05 V (*vs.* Ag/AgCl), (b) current as a function of lactate concentration, and (c) Hanes–Woolf plot for the optimized electrode. Line of fit: 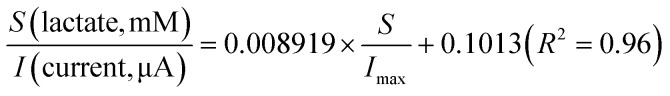.

The lactate sensing performance of the optimized electrode (CP–(LOx–PEGDGE)_4_) was evaluated under a constant potential of −0.05 V (*versus* Ag/AgCl) using CA. Fig. 8(a) illustrates the current response *vs.* time at varying lactate concentrations. With lactate present, the current response decreased over time, stabilizing within 60 s, whereas no current was detected in its absence. The average currents recorded between 60 and 80 s were calculated and plotted against the lactate concentration, as depicted in Fig. 8(b). With increasing lactate concentration, a corresponding increase in current generation was observed. The peak current generation occurred at a lactate concentration of 50 mM and decreased at higher concentrations, a trend typically seen when enzyme saturation occurs at high substrate concentrations.^46^ Typically, enzyme reactions experience a rise in reaction rate with increased substrate concentration until the enzyme becomes saturated with the substrate, leading to a plateau in reaction rate.^47^

The apparent Michaelis–Menten kinetic parameter was determined using a Hanes–Woolf plot (Fig. 8(c)), yielding *K*^app^_m_ and *I*_max_ values of 11.4 mM and 112 μA, respectively. The *K*^app^_m_ obtained here is considerably higher than those reported for free *Aerococcus viridian* LOx (0.1–0.5 mM)^48–50^ and also exceeds those of immobilized LOx (0.2–2.6 mM).^51–53^ This elevation in *K*^app^_m_ likely arises from conformational changes induced by enzyme immobilization and the diffusion restriction imposed by the multi-layer LOx–PEGDGE, which creates a barrier limiting substrate access to the enzymatic matrix.^51^ Importantly, the higher *K*^app^_m_ indicates that substrate saturation occurs only at elevated lactate concentrations, thereby extending the linear detection range compared with conventional LOx-based electrodes. This characteristic enhances the applicability of the optimized electrode for monitoring lactate across a wide concentration range. These results underline the optimized electrode's effective response to changes in lactate levels, highlighting its strong performance in lactate detection. Therefore, this optimized electrode presents significant potential for incorporation into portable lactate sensing devices.

### 3.6 Reusability of optimized electrode

The applied potential was set at −0.1 V because the peak potential of lactate oxidation slightly varied between experiments, and under the reusability test conditions the electrode exhibited a more stable and reproducible current response at −0.1 V compared to −0.05 V during a CA experiment. This adjustment was not intended to redefine the optimal potential universally, but rather to ensure reliable comparison across repeated cycles.

**Fig. 9:**
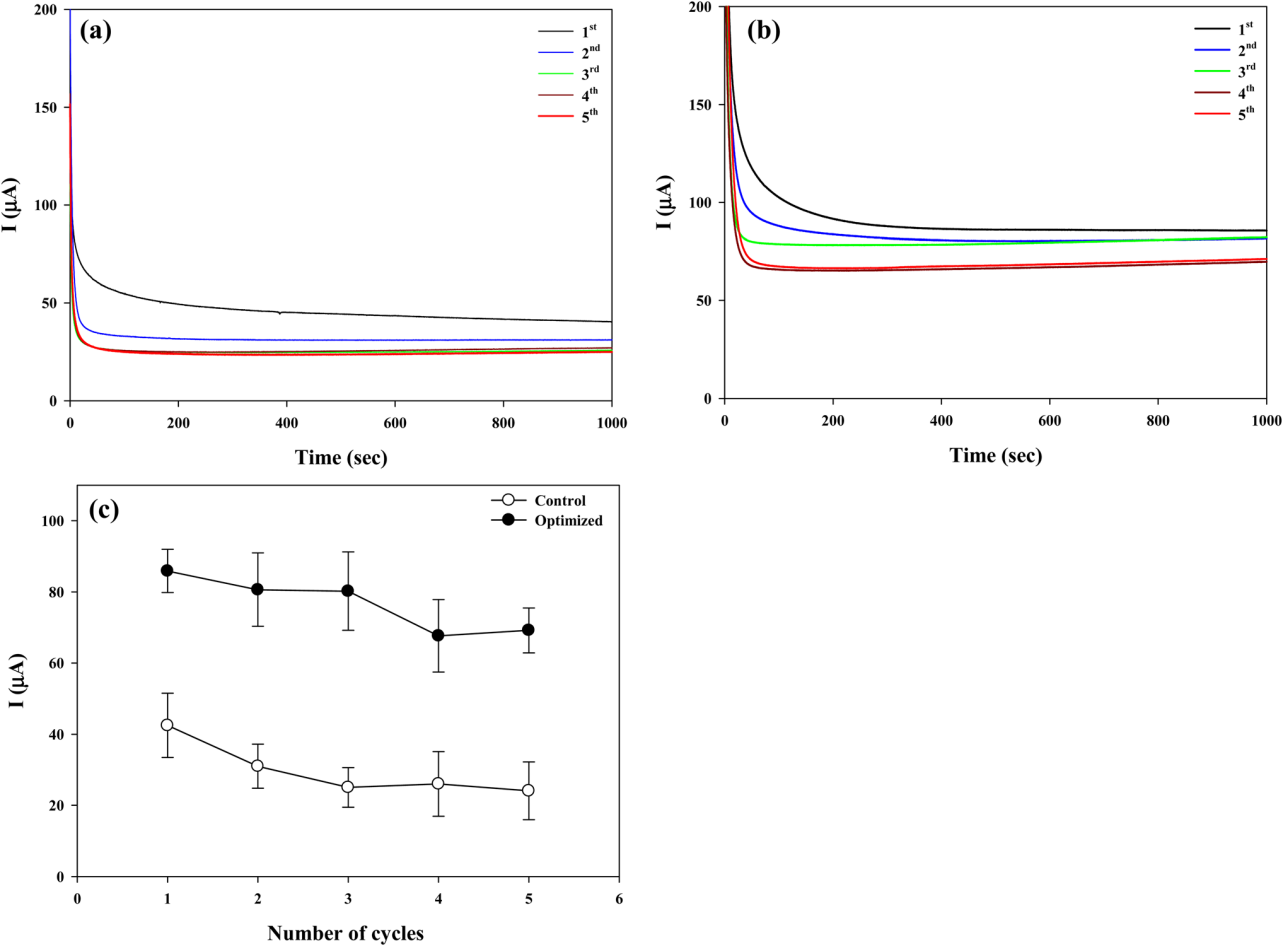
Chronoamperometric current response of (a) control (CP–LOx–PEGDGE) and (b) optimized electrode (CP–(LOx–PEGDGE)_4_) along with (c) the average current values of the electrodes for five repeated cycles. Chronoamperometric analyses were performed in 10 mM PBS (pH = 7.0) supplemented with 1 mM PMS and 50 mM lactate at an applied potential of −0.1 V (*vs.* Ag/AgCl). The electrode was washed with deionized water before reuse, followed by immersion in fresh solution.

A single layer of CP–LOx–PEGDGE was tested as a control electrode for comparison. Both electrodes reached steady-state current responses within 600 s and maintained these levels throughout the duration of each testing cycle, as illustrated in Fig. 9(a) and (b). For each cycle, the currents recorded between 600 and 800 s were integrated and divided by the 200 s production duration to calculate the current output of the electrode. Fig. 9(c) presents the average current responses of the two electrodes. In the first cycle, the optimized electrode (CP–(LOx–PEGDGE)_4_) generated approximately 86 μA, compared to 42 μA from the control (CP–LOx–PEGDGE), indicating a significantly higher (two-fold) current output from the optimized electrode. The current response of both electrodes gradually decreased over three repeated cycles, with no significant further decrease observed after the fourth cycle. However, the rate of decrement differed between the electrodes. In the second cycle, the current of the optimized electrode decreased by 5.8%, while the control electrode showed a 26% reduction. In the third cycle, the steady current for the optimized and control electrodes decreased by approximately 7% and 41%, respectively, from their initial values. In contrast, during the fourth and fifth cycles, the optimized electrode showed decreases of 22% and 19%, while the control exhibited more substantial reductions of 39% and 46%, respectively.

Compared with newly fabricated electrodes (Fig. 4), both electrodes exhibited slight alterations in surface morphology after five repeated cycles (Fig. S1). In the single-layer electrode, the LOx–PEGDGE film displayed pronounced disruption, with cracks, peeling, and exposed carbon domains. These changes were accompanied by an increase in the O/C ratio from 0.186 to 0.245, possibly indicating localized oxidative modification of newly exposed carbon areas. This suggests that the failure mechanism is dominated by the mechanical fragility of the thin coating, with oxidative processes further accelerating degradation. By contrast, the optimized electrode maintained more robust surface coverage, preserving the continuity of the coating after five cycles. Although partial erosion and densification of the outer layers were evident, the internal composite remained intact, sustaining its film-like morphology. This was corroborated by a reduction in the O/C ratio (from 0.464 to 0.381), indicative of partial loss of oxygen-rich components accompanied by relative re-exposure of the carbonaceous substrate. Nevertheless, the optimized electrode retained a higher oxygen content and more compact structure than its single-layer counterpart, indicating improved resistance to structural degradation during repeated use.

These results clearly demonstrate the superior stability of the optimized electrode compared to the control, indicating that even within five repeated cycles the relative stability of the electrode could be sufficiently verified. Nonetheless, extended long-term reusability tests will be considered in future work.

## 4. Conclusion

In this study, response surface analysis was employed to optimize the amount of LOx–PEGDGE attached to the surface of carbon paper. Among the tested configurations, the optimized electrode (CP–(LOx–PEGDGE)_4_) exhibited the highest electrochemical and biochemical performance. The optimization process revealed that stable enzyme binding to the carbon paper surface significantly enhanced the electrode's stability, enabling repeated use over multiple cycles. This finding underscores the potential of the optimized electrode for practical applications in biosensing and bioelectrocatalysis, offering both high efficiency and durability.

## Author contributions

KS: methodology, formal analysis, writing—original draft. SV: additional experiments and analysis. KBM: formal analysis, writing—revised manuscript. GL: statistical analysis. CK: supervision, conceptualization, writing—review & editing.

## Conflicts of interest

There are no conflicts of interest to declare.

## Supplementary Material

RA-015-D5RA07173A-s001

## Data Availability

All data generated or analyzed during this study are included in this manuscript file. Supplementary information: Fig. S1. See DOI: https://doi.org/10.1039/d5ra07173a.
