# Supplementary material for: Optimization and characterization of a lactate-oxidase electrode
Source: RSC Adv. 2025 Nov 4;15(50):42533–44. doi: 10.1039/d5ra07173a (PMC12584134; doi:10.1039/d5ra07173a)
Supplement: RA-015-D5RA07173A-s001 [file RA-015-D5RA07173A-s001.pdf]

Supplementary Information (SI) for RSC Advances.
This journal is © The Royal Society of Chemistry 2025

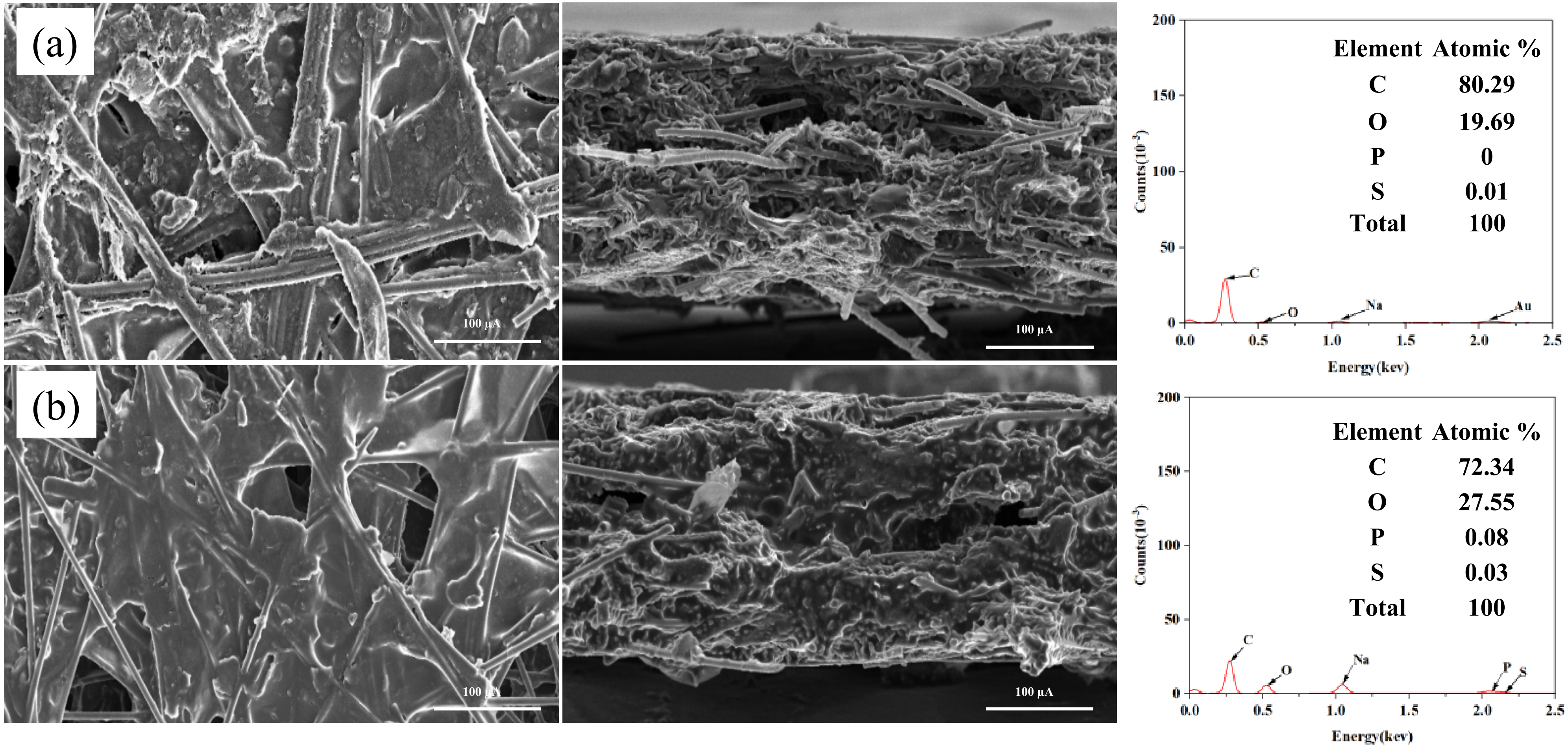

Fig. S1 FE-SEM images and EDS mappings of the tops and cross sections of (a) CP with one layer of LOx-PEGDGE, and (b) CP with four layers of LOx-PEGDGE (optimized electrode) after five repeated cycles.
